# Balanced oral pathogenic bacteria and probiotics promoted wound healing via maintaining mesenchymal stem cell homeostasis

**DOI:** 10.1186/s13287-020-1569-2

**Published:** 2020-02-14

**Authors:** Nannan Han, Lu Jia, Lijia Guo, Yingying Su, Zhenhua Luo, Juan Du, Shenghui Mei, Yi Liu

**Affiliations:** 1 Laboratory of Tissue Regeneration and Immunology and Department of Periodontics, Beijing Key Laboratory of Tooth Regeneration and Function Reconstruction, School of Stomatology, Capital Medical University, Tian Tan Xi Li No.4, Beijing, 100050 People’s Republic of China; 2 Department of Orthodontics, School of Stomatology, Capital Medical University, Beijing, People’s Republic of China; 3 Beijing Tiantan Hospital, Capital Medical University, Beijing, People’s Republic of China

**Keywords:** Oral microbiomes, MSCs, Wound healing, *P. gingivalis*, *L. reuteri*

## Abstract

**Objectives:**

The homeostasis of oral pathogenic bacteria and probiotics plays a crucial role in maintaining the well-being and healthy status of human host. Our previous study confirmed that imbalanced oral microbiota could impair mesenchymal stem cell (MSC) proliferation capacity and delay wound healing. However, the effects of balanced oral pathogenic bacteria and probiotics on MSCs and wound healing are far from clear. Here, the balance of pathogenic bacteria *Porphyromonas gingivalis* and probiotics *Lactobacillus reuteri* extracts was used to investigate whether balanced oral microbiota modulate the physiological functions of MSCs and promote wound healing.

**Methods:**

The effects of balanced pathogenic bacteria *P. gingivalis* and probiotics *L. reuteri* extracts on gingival MSCs (GMSCs) were tested using the migration, alkaline phosphatase activity, alizarin red staining, cell counting kit-8, real-time PCR, and western blot assays. To investigate the role of balanced pathogenic bacteria *P. gingivalis* and probiotics *L. reuteri* extracts in the wound of mice, the wounds were established in the mucosa of palate and were inoculated with bacteria every 2 days.

**Results:**

We found that the balance between pathogenic bacteria and probiotics enhanced the migration, osteogenic differentiation, and cell proliferation of MSCs. Additionally, local inoculation of the mixture of *L. reuteri* and *P. gingivalis* promoted the process of wound healing in mice. Mechanistically, we found that LPS in *P. gingivalis* could activate *NLRP3* inflammasome and inhibit function of MSCs, thereby accelerating MSC dysfunction and delaying wound healing. Furthermore, we also found that reuterin was the effective ingredient in *L. reuteri* which maintained the balance of pathogenic bacteria and probiotics by neutralizing LPS in *P. gingivalis*, thus inhibiting inflammation and promoting wound healing.

**Conclusions:**

This study revealed that the homeostasis of oral microbiomes played an indispensable role in maintaining oral heath, provided hopeful methods for the prevention and treatment of oral diseases, and had some referential value for other systemic diseases caused by dysfunction of microbiota and MSCs.

## Introduction

The oral cavity, as an extension of an external body part, is actually colonized by billions of bacteria, fungi, and viruses, now known as oral microbiomes. It is fairly well known that oral microbial community includes pathogenic bacteria and probiotics, and that homeostasis of oral microbiomes plays a crucial role in maintaining the well-being and healthy status of human host [1–6]. Strong evidence has shown that once the balance of oral microbiota is broken, predominant pathogens can lead to a variety of oral diseases including periodontitis, caries, odontogenic infections, and oral mucositis [7–9]. Furthermore, several studies seem to suggest that *Porphyromonas gingivalis* (*P. gingivalis*) may be a driving factor in the development of oral and digestive cancer, such as oral squamous cell carcinoma and intestinal and pancreatic cancer [10, 11]. It is now widely accepted that alterations in the dysbiosis of the oral microbiota may influence the normal host-microbe crosstalk, thus resulting in a higher risk for the onset of diseases. Probiotics are a kind of microorganisms which are beneficial to the health of the host and can produce health care effects when given to the host in proper dose. Probiotics, including *Lactobacillus*, *Bifidobacteria*, *Escherichia coli*, and *Enterococcus faecalis*, mainly keep the health of body by maintaining the balance of host bacteria, secreting antimicrobial substances, and modulating immune response [12]. Previous studies have shown that probiotics can inhibit the proliferation and adhesion of oral cariogenic bacteria by colonizing in oral cavity and producing metabolites. In addition, related studies have found that oral probiotics can also improve the halitosis by adjusting oral flora and reducing volatile sulfides. Oral candidiasis is a common oral mucosal infectious disease. In vitro experiments have demonstrated that probiotics can inhibit the proliferation, growth, and adhesion of *Candida albicans* and reduce the formation of biofilm [3, 13]. Therefore, the balance between pathogenic bacteria and probiotics is very indispensable to maintain oral health.

Oral wounds are commonly caused by surgical excision of lesions, vulnus, recurrent ulcer, and radiation injury, and usually accompanied by oral mucosal and soft tissue defects, which can lead to scar formation and tissue adhesion. It has been reported that wound healing process is delayed as a result of disturbances of microbiota adhesion at the sites of wounds [14–17]. Mesenchymal stem cells (MSCs) are considered as a promising method that has the potential to promote tissue regeneration and wound healing due to the multilineage differentiation and self-renewal properties [18–21]. Our previous study found that in an environment of oral microecological imbalance, the function of oral gingival and palatal MSCs was impaired, which played an important role in the repair of oral soft tissue damage, and the healing speed of oral soft tissue was significantly slowed down [22]. However, the effects of the balance between oral pathogenic bacteria and probiotics on the physiological function of MSCs and wound healing are still unclear.

In this work, we investigated the role of the balance of oral pathogenic bacteria and probiotics in the regulation of MSCs’ potentials and wound healing. Our results revealed that simulating the balance between oral pathogenic bacteria and probiotics with sonicated extracts from *L. reuteri* and *P. gingivalis* could activate the migration, osteogenic differentiation, and proliferation of GMSCs in vitro. Furthermore, we also found that reuterin was the effective ingredient in *L. reuteri* which maintained the balance of oral pathogenic bacteria and probiotics by neutralizing LPS in *P. gingivalis*. Additionally, the local inoculation of the mixture of *L. reuteri* and *P. gingivalis* restored the process of wound healing in mice. Our findings are based on the concept of the balance of oral microecology, which provides promising ideas and methods for the prevention and treatment of oral diseases, and can be used for reference in other systemic diseases caused by dysfunction of microbiota and MSCs.

## Materials and methods

### Animals

Eight-week-old female C57BL/6 mice were gained from SPF Biotechnology Company (Beijing, China). Mice were raised under the standard conditions following the Animal Care and Use Committee of Capital Medical University. All animal researches were abided by the rules approved by the Beijing Stomatological Hospital, Capital Medical University (Ethical Committee Agreement, Beijing Stomatological Hospital Ethics Review No. KQYY-201710-001).

### Cell cultures

Gingival tissues were isolated from C57BL/6 mice. Solutions of 75% ethanol and phosphate-buffered saline (PBS) were used to disinfect and rinse the tissues. After that, gingival tissues were digested by a solution of 3 mg/ml collagenase type I (Sigma-Aldrich, USA) and 4 mg/ml dispase (Sigma-Aldrich, USA) for 1 h at 37 °C. A 70-μm strainer (Falcon, USA) was used to filter dissociated GMSC suspensions. GMSCs were cultivated in a humidified incubator under 5% CO_2_ at 37 °C in DMEM alpha modified Eagle’s medium (Invitrogen, USA), renewal with 20% fetal bovine serum (FBS; Invitrogen), 100 μg/ml streptomycin, 100 U/ml penicillin, and 2 mmol/l glutamine (Invitrogen).

### Bacterial strain culture and preparation of bacterial extracts

*Porphyromonas gingivalis* (*P. gingivalis*) ATCC 33277 and *Lactobacillus reuteri* (*L. reuteri*) ATCC 11284 were grown on agar plates at 37 °C in an anaerobic chamber with MRS (AOBOX, Beijing) for 5–7 days. For experiments in vitro on GMSCs, *Lactobacillus reuteri* cells were scraped and resuspended in 10 ml phosphate-buffered solution (PBS), sonicated (30 min, 300 W, 10 s sonication, and 10 s pause) with ultrasonic crushing apparatus (JYD-150, China) on an ice bath. The sonicated preparation was centrifuged at 14,000 rpm for 20 min at 4 °C and discarded the supernatants; the weight of precipitates was taken and diluted with sterile deionized water (bacterial extracts) [23]. Bacterial extracts reached a certain concentration and filtered with 0.22-μm strainer (Falcon, USA), stored at 20 °C until used. For animal experiments in vivo, bacteria (*P. gingivalis* and *L. reuteri*) should be cultured way in advance. On the day of treatment, bacteria were harvested by centrifugation, washed three times with PBS, and quantified using spectrophotometer. (the number of *P. gingivalis* at OD of 0.8 was approximately 1 × 10^9^/ml). Bacteria were resuspended in 2% methylcellulose (Sigma Aldrich, USA) (2 × 10^9^/100 μl/mouse).

### Cell scratch migration assays

GMSCs were cultured on six-well plates as confluent monolayers, a cross wound was scratched using a 200-μl pipet tip (Axygen® Corning, NY, USA), and then, cells were cultured in fresh culture media without fetal bovine serum. To observe the extent of wound migration, images were taken using microscopy at baseline (0 h), 24 h, 48 h, and 72 h after scratching. The void area (VA) of wound was measured by Image-Pro (National Institutes of Health, USA), and the height and the relative width were calculated (Area% = VA/height).

### Real-time reverse transcriptase-polymerase chain reaction

Total RNA was isolated from GMSCs using TRIzol reagents (Invitrogen, USA) and reverse transcribed into cDNA following the manufacturer’s protocol (Takara, Dalian, China). Then, real-time RT-PCR reactions were performed using the SYBR Premix Ex TaqTM (Takara, Dalian, China) and an Icycler iQ Multi-colour Real-time RT-PCR Detection System. The primers for specific genes are listed in Additional file 1: Table S1.

### Alkaline phosphatase and alizarin red detection

To detect the abilities of GMSC osteogenesis and mineralization, we used alizarin red staining and alkaline phosphatase activity assays according to the StemPro osteogenesis differentiation kit (Invitrogen, USA) and the manufacturer’s protocol of ALP activity (Sigma-Aldrich, USA).

### Oil Red O staining analysis

GMSCs were cultivated on six-well plates and treated with *P. gingivalis* and *L. reuteri* sonic extracts respectively in lipogenic induction medium (Invitrogen, USA) for 1 month. Then, the medium was removed, added with 2 ml of 10% formalin, and incubated for 10 min at room temperature. Cells were washed with 2 ml of 60% isopropanol for 5 min, and let the cells dry completely. One milliliter of Oil Red O working solution was added and incubated at RT for 10 min. Images were acquired under the microscope for analysis. Oil Red O dye (Sigma, USA) was eluted by adding 1 ml of 100% isopropanol and incubated for 10 min with gentle shaking. OD value was measured at 500 nm using 100% isopropanol as blank.

### Western blot analysis

GMSCs were lysed using RIPA buffer. The details of method for western blot were depicted as previously [24]. The expressions of protein were detected using anti-NLRP3 (1:300, Abcam), and β-actin (1:2000, Abcam) was used as an internal control. The secondary antibodies were obtained from the commercial companies: anti-mouse IgG (1:2000, Abcam) and anti-rabbit IgG (1:5000, Abcam).

### 16S rRNA sequencing and analysis

An antibiotic cocktail of 1300 mg/l of metronidazole and 660 mg/l of levofloxacin in the drinking water was used to remove oral bacteria in mice for 2 weeks. After that, the bacteria of oral mucosa of mice were scraped with cotton swab and analyzed as previously [22]. UCLUST was used to divide the unique sequence set into operational classifiers (OTUs) under 97% recognition threshold. Student’s *t* test was implemented to evaluate alpha and beta diversity. The Kruskal-Wallis test was used to test the significance of classified variables.

### Wound healing mouse model

C57BL/6 mice were anesthetized by intraperitoneal injection of 1% chloral hydrate using 1 ml syringe. Chloral hydrate was injected based on the weight of the mice; the delivered dose is chloral hydrate 400 mg/kg. The wounds were established in the mucosa of palate, and the region of wound was from the mesial margin of the first molar to the distal of the third molar; full thickness mucosa was removed. Mice with wound were randomly distributed to four groups: incubation of *P. gingivalis* group, incubation of *L. reuteri* group, incubation of *P. gingivalis* and *L. reuteri* group, or untreated group. The wounds were inoculated with bacteria every 2 days. To observe the extent of wound healing, mice were sacrificed after incubation for 7 and 14 days. Stereomicroscopy and Image J were used to measure the area of wound healing. Then, the maxillary palates were fixed in 4% paraformaldehyde for 48 h, and all samples were decalcified with buffered 10% EDTA and embedded in paraffin. Then, all samples were deparaffinized and stained with hematoxylin and eosin.

### Statistics

The SPSS10 software was used to analyze all statistical computations. Statistical significance was identified by Student’s *t* test, Duncan’s test, or one-way ANOVA, with a *P* ≤ 0.05 regarded as significant.

## Results

### The balance between pathogenic bacteria and probiotics restored GMSC migration

**Fig. 1:**
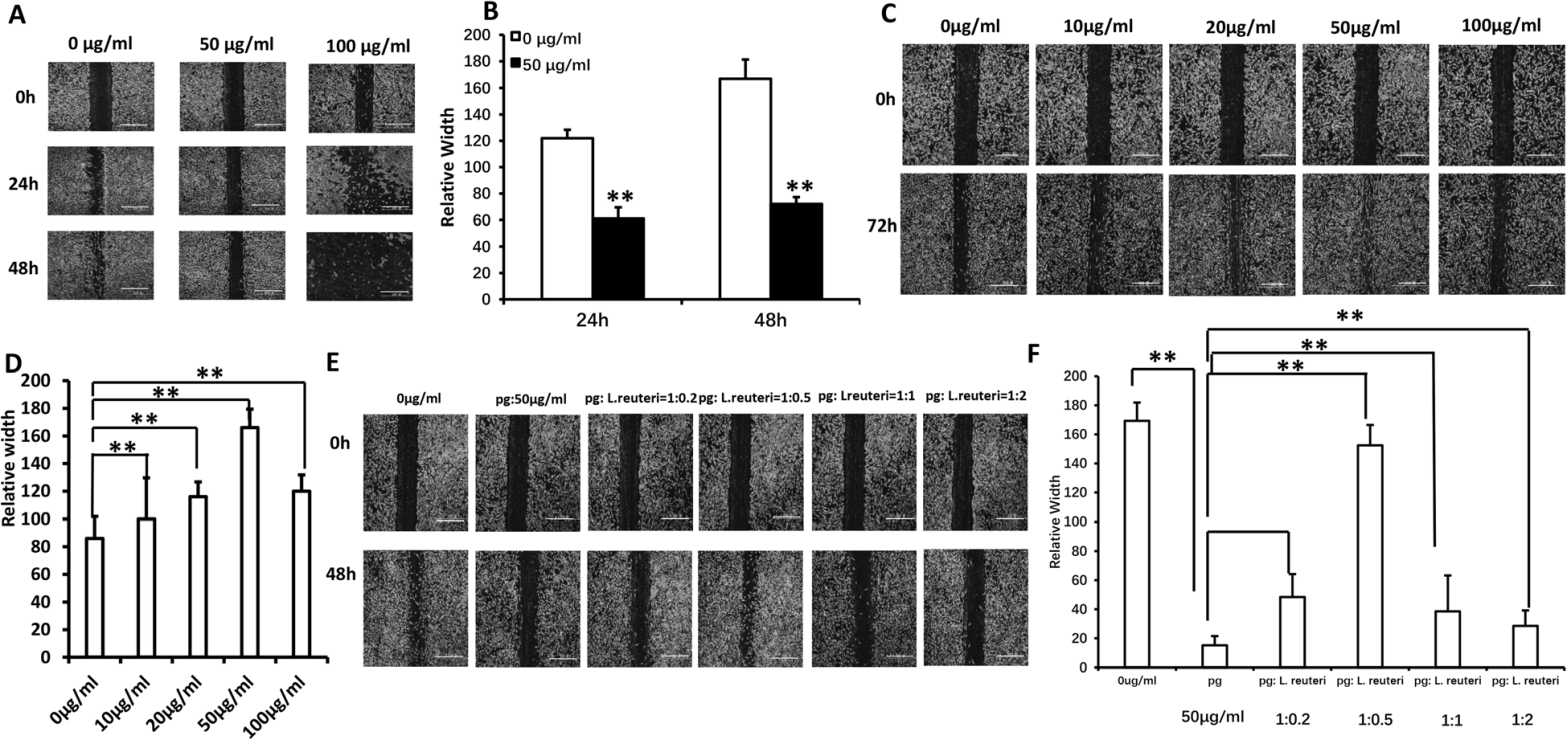
The balance between pathogenic bacteria and probiotics restored GMSC migration. **a**, **b** The cell scratch migration assay results showed that 50 μg/ml *P. gingivalis* sonicated extracts significantly inhibited GMSC migration and screened 50 μg/ml *L. reuteri* sonicated extracts markedly promoted GMSC migration (**c**, **d**). **e**, **f** The cell scratch migration results demonstrated that the ratio of *P. gingivalis* bacteria extracts to *L. reuteri* sonicated extracts was 1 to 0.5 (1, 50 μg/ml *P. gingivalis* sonicated extracts; 0.5, 25 μg/ml *L. reuteri* sonicated extracts), which restored GMSC migration. Scale bar 100 μm. One-way ANOVA was used to verify statistical significance. Error bars represent SD (*n* = 3). *P ≤ 0.05; **P ≤ 0.01

To investigate the balance of oral pathogenic bacteria and probiotics in vitro, we performed scratch-simulated wound migration assay in GMSCs. In the first place, different concentrations of the *P. gingivalis* sonicated extracts were added to the cells; according to cell scratch migration assay results, we found that 50 μg/ml *P. gingivalis* sonicated extracts significantly inhibited GMSC migration at 24 h and 48 h (Fig. 1a, b). Secondly, to investigate the effect of probiotics on the migration of GMSCs, we discovered that 50 μg/ml *L. reuteri* sonicated extracts markedly promoted GMSC migration (Fig. 1c, d). Finally, according to the above screened concentration of *P. gingivalis* and *L. reuteri* sonicated extracts, respectively, on the basis of 50 μg/ml *P. gingivalis* sonicated extracts, we mixed different proportions of pathogenic bacteria and probiotics into the GMSCs; the cell scratch migration results demonstrated that the ratio of *P. gingivalis* bacteria extracts to *L. reuteri* sonicated extracts was 1 to 0.5 (1, 50 μg/ml *P. gingivalis* sonicated extracts; 0.5, 25 μg/ml *L. reuteri* sonicated extracts), which restored GMSC migration (Fig. 1e, f). Next, the ratio of 1 to 0.5 was used to mimic the balance of oral pathogenic bacteria and probiotics in all experiments in vitro.

### The balance between pathogenic bacteria and probiotics restored the function of GMSCs

**Fig. 2:**
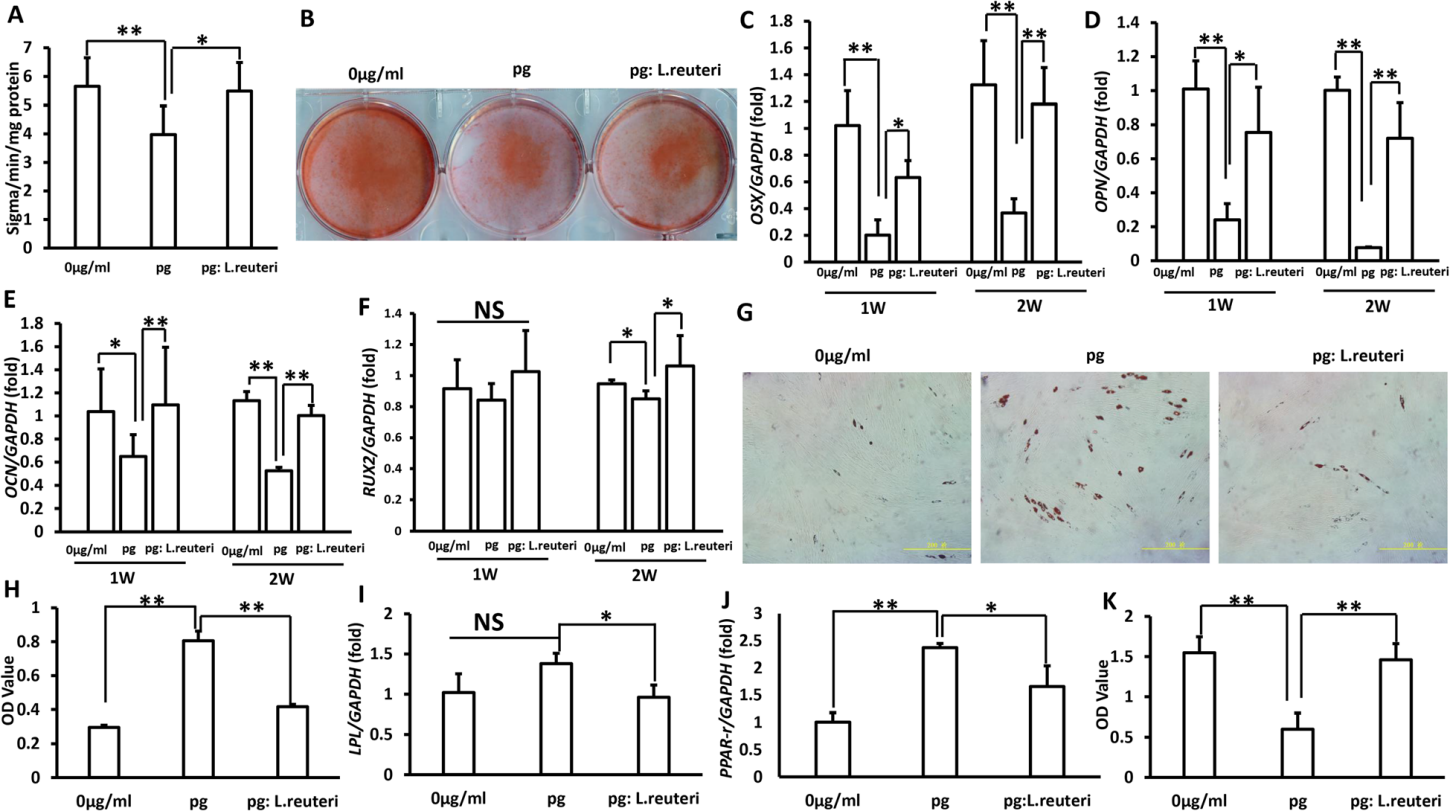
The balance of oral pathogenic bacteria and probiotics restored the functions of GMSCs. **a** ALP activity. **b** Alizarin red staining. **c**–**f** The expression of the crucial transcription factors for modulating osteogenic differentiation: OSX, OPN, OCN, and RUNX2 expressions were increased after restoring the balance of oral microbiota. **g**, **h** Oil Red O staining analysis. **i**, **j** Real-time RT-PCR results demonstrated that the key adipogenic transcription factors PPAR-γ and LPL were decreasingly expressed after the mixture of *P. gingivalis* bacteria extracts and *L. reuteri* sonicated extracts treatment, and GAPDH was used as an internal control. **k** Cell counting kit-8 assay results. Student’s *t* test and one-way ANOVA were used to test statistical significance. Error bars represent SD (*n* = 3). *P ≤ 0.05; **P ≤ 0.01

To test the effect of the balance between pathogenic bacteria and probiotics on GMSCs, the cells were treated with 50 μg/ml *P. gingivalis* sonicated extracts and the mixture of *P. gingivalis* bacteria extracts and *L. reuteri* sonicated extracts. The osteogenic differentiation abilities of GMSCs were evaluated using ALP activity assay, the cells were transduced in osteogenic-inducing medium for 5 days, and we found that the ratio of *P. gingivalis* sonicated extracts to *L. reuteri* sonicated extracts was 1 to 0.5, which rescued the ALP activity of GMSCs (Fig. 2a). Three weeks after osteogenic induction, the alizarin red staining assay results showed that the balance between pathogenic bacteria and probiotics rescued the osteogenic differentiation capacity which was impaired by *P. gingivalis* sonicated extract treatment (Fig. 2b). In addition, to further test the impact of microbiome balance on osteogenic GMSCs, we performed real-time RT-PCR to verify the expression of the crucial transcription factors for regulating osteogenic differentiation: *OSX*, *OPN*, *OCN*, and *RUNX2*, and our results demonstrated that the ratio of *P. gingivalis* sonicated extracts to *L. reuteri* sonicated extracts was 1 to 0.5, which restored the expressions of *OSX*, *OPN*, *OCN*, and *RUNX2* (Fig. 2c–f). Oil Red O staining analysis results showed that the balance between pathogenic bacteria and probiotics inhibited the GMSC adipogenic differentiation caused by *P. gingivalis* sonicated extracts (Fig. 2g, h). Meanwhile, real-time RT-PCR results demonstrated that the key adipogenic transcription factors *PPAR-γ* and *LPL* were decreasingly expressed after the mixture of *P. gingivalis* bacteria extract and *L. reuteri* sonicated extract treatment (Fig. 2i, j). The cell counting kit-8 assay results showed that he ratio of *P. gingivalis* sonicated extracts to *L. reuteri* sonicated extracts was 1 to 0.5, which rescued the proliferation capacity of GMSCs compared to the *P. gingivalis* sonicated extract group (Fig. 2k). These findings elucidated that the balance between pathogenic bacteria and probiotics could maintain the functions of GMSCs.

### The oral predominant pathogenic bacteria in mice decreased after antibiotic treatment

**Fig. 3:**
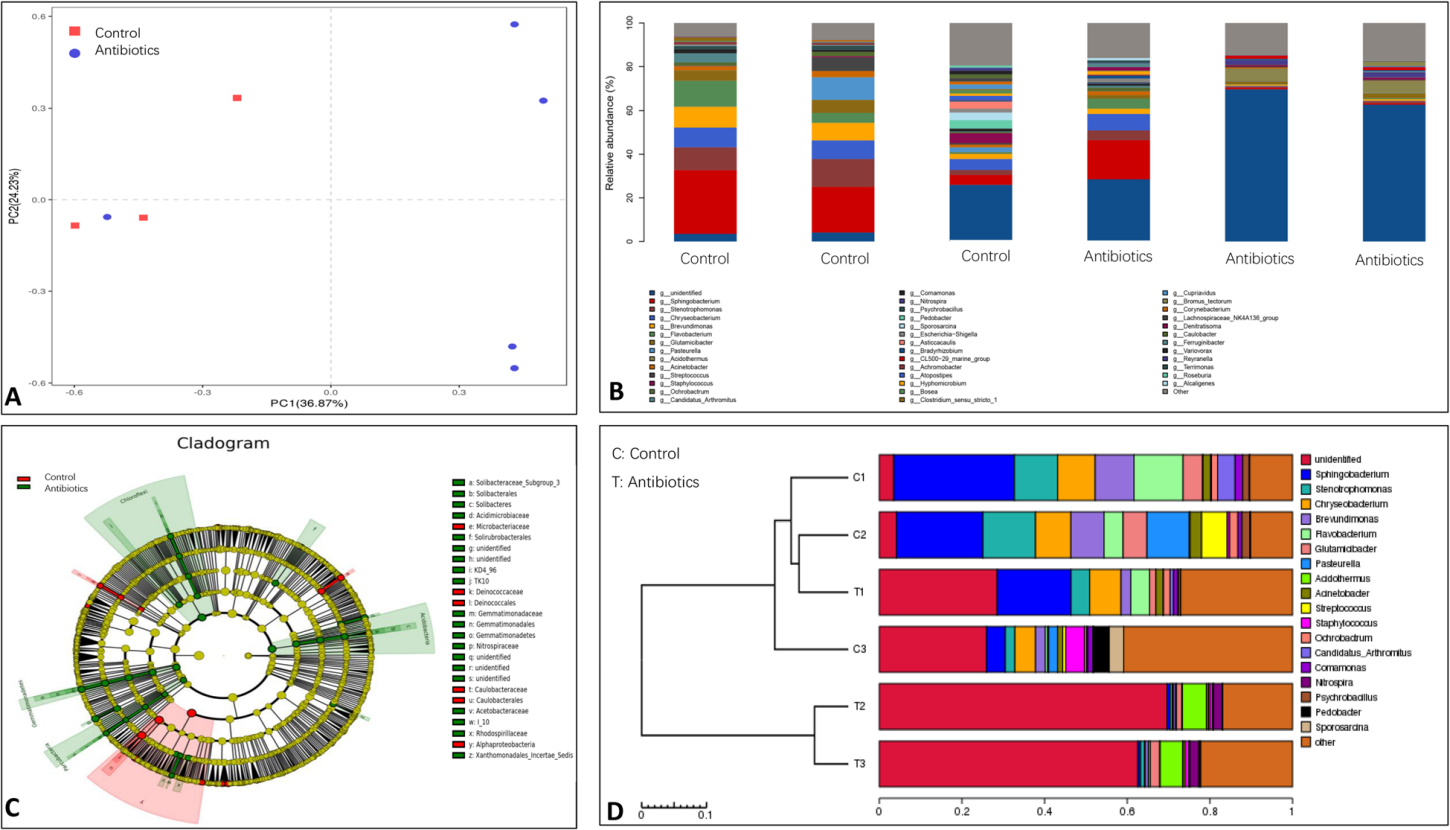
The oral predominant pathogenic bacteria in mice decreased after antibiotic treatment. **a** Beta diversity was calculated by unweighted UniFrac, and the distances were plotted with principal coordinate analysis (PCA). **b** Barplot analysis further revealed that the bacterial relative abundance was significantly decreased in antibiotic treatment group compared with control group. **c** LefSe analysis results showed that the predominant pathogenic bacteria decreased after antibiotic treatment. **d** UPGMA analysis

To analyze the variation of oral bacterial communities in mice after antibiotic treatment, we collected oral microbiota from oral mucosa tissue samples, and 16S rRNA gene sequencing results demonstrated that the differences of a general discrete clustering pattern of bacterial taxa were observed between mice with antibiotic treatment and control group (Fig. 3a). Barplot analysis further revealed that the bacterial relative abundance was significantly decreased in antibiotic treatment group compared with control group (Fig. 3b). LefSe analysis results showed that the predominant pathogenic bacteria decreased after antibiotic treatment (Fig. 3c). On the basis of UPGMA analysis, we further demonstrated that the microbial communities were reduced in antibiotics group (Fig. 3d), and these findings suggested that the predominant pathogenic bacteria decreased after antibiotic treatment.

### The balance of pathogenic bacteria and probiotics promoted wound healing process in mice

**Fig. 4:**
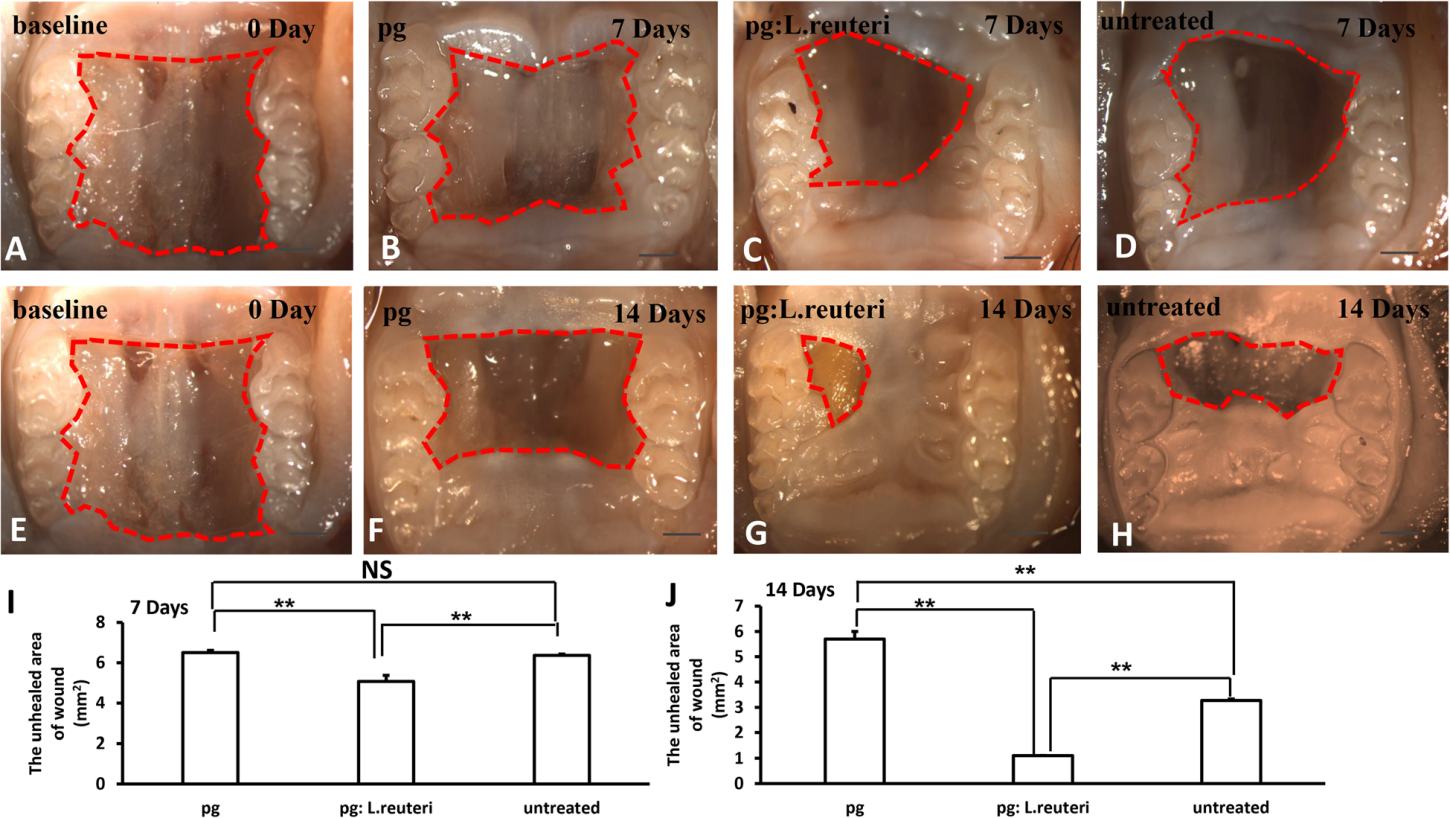
The balance of oral pathogenic bacteria and probiotics promoted wound healing process in mice. **a**, **e** Macroscopic observation showed that wound healing model of palate was established in mice after antibiotic treatment. **b**, **f** The inoculation of *P. gingivalis* delayed wound healing after 7 and 14 days. **c**, **g** The inoculation of the mixture of *L. reuteri* and *P. gingivalis* group promoted wound healing compared with untreated group (**d**, **h**). **i**, **j** Quantitative analysis of the unhealed area of wound after inoculation for 7 and 14 days, respectively. Scale bar 1 mm. Error bars represent SD (*n* = 6). Red dotted line, the unhealed area of wound. *P ≤ 0.05; **P ≤ 0.01

To further verify the effect of the balance of oral pathogenic bacteria and probiotics on wound, we established wound healing model of palate in mice after antibiotic treatment (Fig. 4a, e). The wounds were inoculated with either *P. gingivalis* (2 × 10^9^ CFU in 2% methylcellulose 200 μl) or the mixture of *L. reuteri* and *P. gingivalis*, or untreated group. Each group was inoculated once every 2 days. The mice were sacrificed after inoculation for 7 and 14 days, and the area of wound healing was observed by stereomicroscopy and measured using Image J. We discovered that inoculation of the mixture of *L. reuteri* and *P. gingivalis* group promoted wound healing (Fig. 4c, g), while the wound healing process was delayed in the inoculation of *P. gingivalis* group (Fig. 4b, f), and the wound healing in the untreated group was slower than the mixture of *L. reuteri* and *P. gingivalis* group (Fig. 4d, h). In addition, we assessed the quantity of the unhealed area of wound; the results showed statistically significant differences in the *P. gingivalis* inoculation group, in the mixture of *L. reuteri* and *P. gingivalis* group, and in the untreated group (Fig. 4i, j), suggesting that the balance of oral microbiome promoted wound healing in mice.

**Fig. 5:**
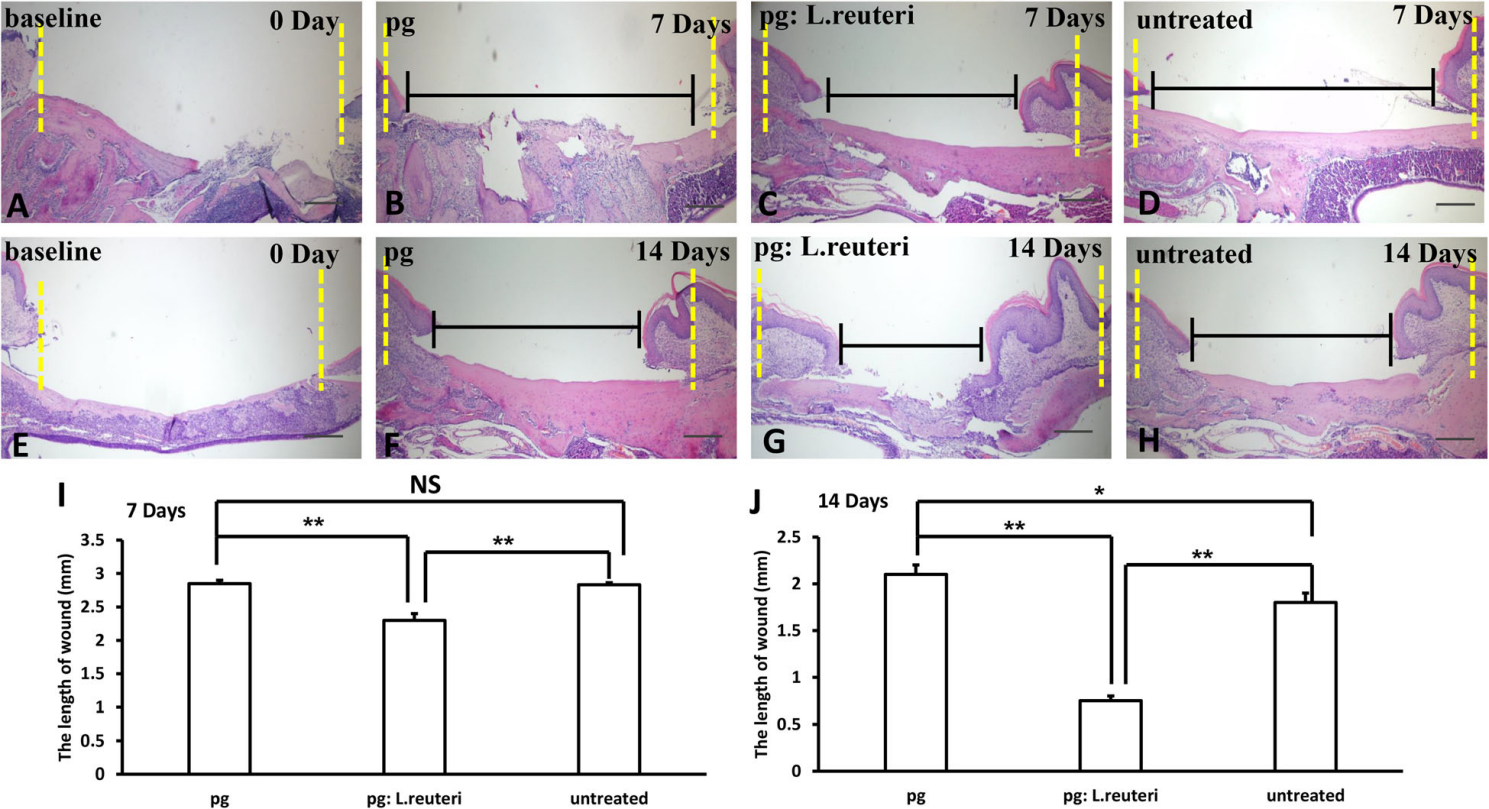
The balance of oral pathogenic bacteria and probiotics promoted wound healing process in mice by histopathological analysis. **a**, **e** H&E staining results showed the original length of wound in mice. The inoculation of the mixture of *L. reuteri* and *P. gingivalis* group (**c**, **g**) markedly accelerated wound healing compared to the *P. gingivalis* group (**b**, **f**) and untreated group (**d**, **h**). **i**, **j** Quantitative analysis of the residual length of wound after inoculation for 7 and 14 days, respectively. Scale bar 50 μm. Error bars represent SD (*n* = 6). Yellow dotted line, the original length of wound. Black line, the length of wound. *P ≤ 0.05; **P ≤ 0.01

Histopathological photomicrograph results further revealed that the inoculation of the mixture of *L. reuteri* and *P. gingivalis* group (Fig. 5c, g) markedly accelerated wound healing compared to the *P. gingivalis* group (Fig. 5b, f) and untreated group (Fig. 5d, h). Furthermore, the length of the wound was measured using Image J, we found that the length of wound was shorter in the mixture of *L. reuteri* and *P. gingivalis* group than in the inoculation of *P. gingivalis* group and untreated group (Fig. 5i, j), and there were significant statistical differences in these three groups. These findings further demonstrated that the balance of oral microbiome played a crucial role in wound healing process.

### LPS increased in *P. gingivalis* and thereby inhibited the functions of MSCs by activating NLRP3 inflammasome

**Fig. 6:**
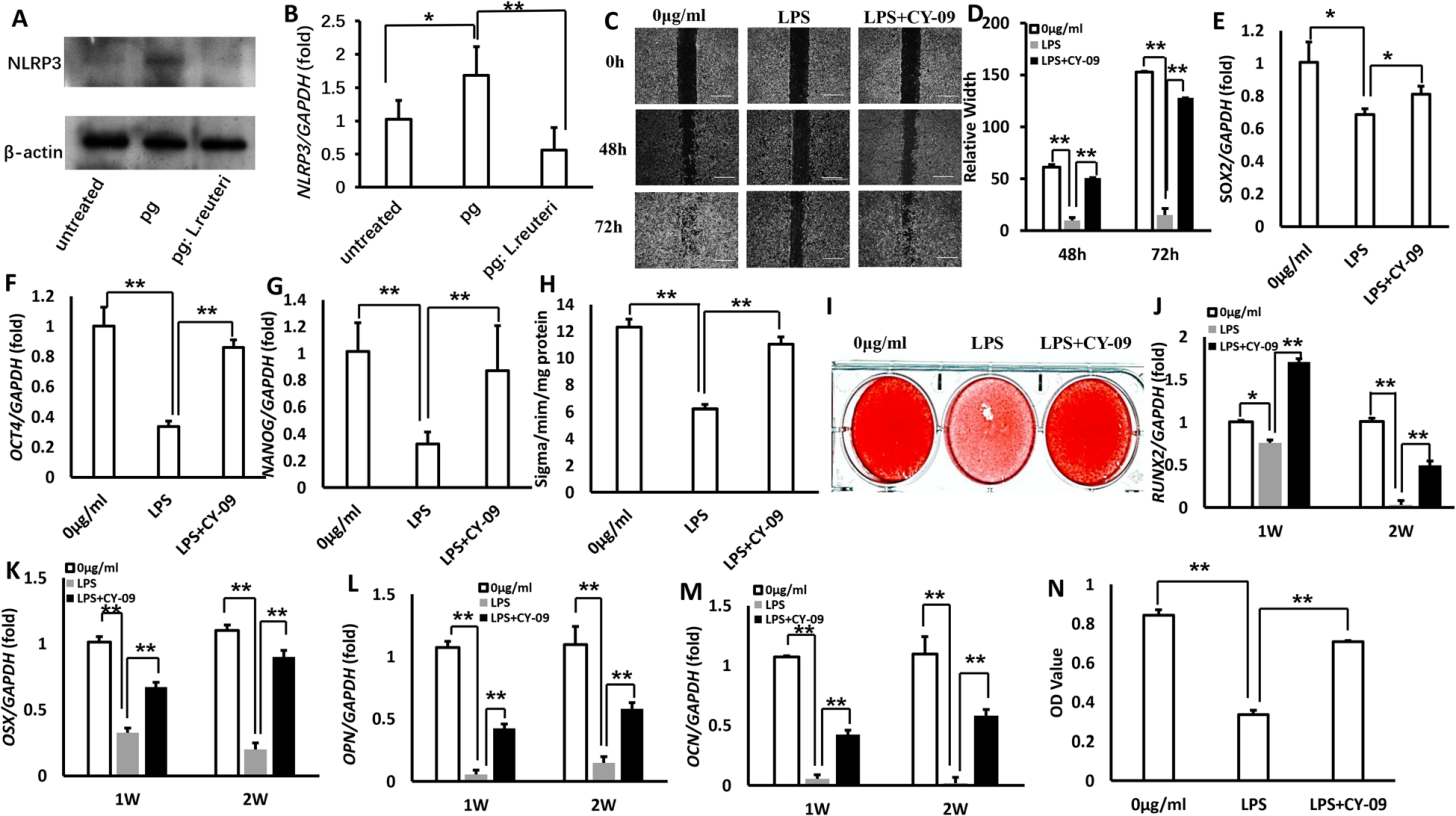
LPS increased in *P. gingivalis* and thereby inhibited the functions of MSCs by activating NLRP3 inflammasome. **a**, **b** Western blot and real-time RT-PCR results revealed that NLRP3 expression was significantly increased in palatal tissues from the inoculation of *P. gingivalis* group compared to the mixture of *L. reuteri* and *P. gingivalis* group, and untreated group. β-actin was used as an internal control in western blot assay. **c**, **d** The cell migration assay results demonstrated that LPS significantly inhibited GMSC migration, and NLRP3 inhibitor CY-09 rescued the capacity of GMSC migration after LPS treatment. **e**–**g** Real-time RT-PCR results showed that LPS inhibited the expression of SOX2, OCT4, and NANOG, and CY-09 restored the capacities of MSCs. **h**, **i** ALP activity assay and alizarin red staining assay. **j**-**m** Real-time RT-PCR results further testified that NLRP3 activation inhibited the expression of the crucial transcription factors for modulating osteogenic differentiation: RUX2, OSX, OPN, and OCN, and CY-09 rescued the functions of osteogenic differentiation. **n** Counting kit-8 assay results showed that CY-09 restored the proliferation potential of GMSCs caused by LPS-induced NLRP3 activation. GAPDH was an internal control. Error bars represent SD (*n* = 3). *P ≤ 0.05; **P ≤ 0.01

It has been reported that *NLRP3* inflammasome played an important role in immune response and a wide variety of disease occurrence [25–27]. To identify if the underlying molecular mechanisms of *P. gingivalis* inhibited the functions of MSCs, we tested the expression of *NLRP3* from the wound tissues of palate in mice after inoculation with bacteria; western blot and real-time RT-PCR results revealed that *NLRP3* expression was significantly increased in the inoculation of *P. gingivalis* group compared to the mixture of *L. reuteri* and *P. gingivalis* group, and untreated group (Fig. 6a, b). Next, we used exogenous LPS as *NLRP3* activator and CY-09 as *NLRP3* inhibitor to test the functions of GMSCs [28]; the cell migration assay results showed that LPS significantly inhibited GMSC migration; however, *NLRP3* inhibitor CY-09 rescued the capacity of GMSC migration after LPS treatment (Fig. 6c, d). This suggested that LPS could activate *NLRP3* inflammasome. To further testify the impact of *NLRP3* activation on mesenchymal stem cell markers, real-time RT-PCR results showed that LPS inhibited the expression of *SOX2*, *OCT4*, and *NANOG*, while CY-09 restored the capacities of MSCs (Fig. 6e–g). ALP activity assay and alizarin red staining assay results confirmed that *NLRP3* activation inhibited ALP activity and mineralization in GMSCs (Fig. 6h, i); moreover, real-time RT-PCR results further testified that *NLRP3* activation inhibited the expression of the crucial transcription factors for modulating osteogenic differentiation: *RUX2*, *OSX*, *OPN*, and *OCN* (Fig. 6j–m), and CY-09 rescued the functions of osteogenic differentiation. In addition, counting kit-8 assay results showed that CY-09 restored the proliferation potential of GMSCs caused by LPS-induced *NLRP3* activation (Fig. 6n). On the basis of these results, we demonstrated that *P. gingivalis* inhibited the functions of MSCs by activating *NLRP3* inflammasome.

### Reuterin maintained the balance of oral pathogenic bacteria and probiotics by neutralizing LPS

**Fig. 7:**
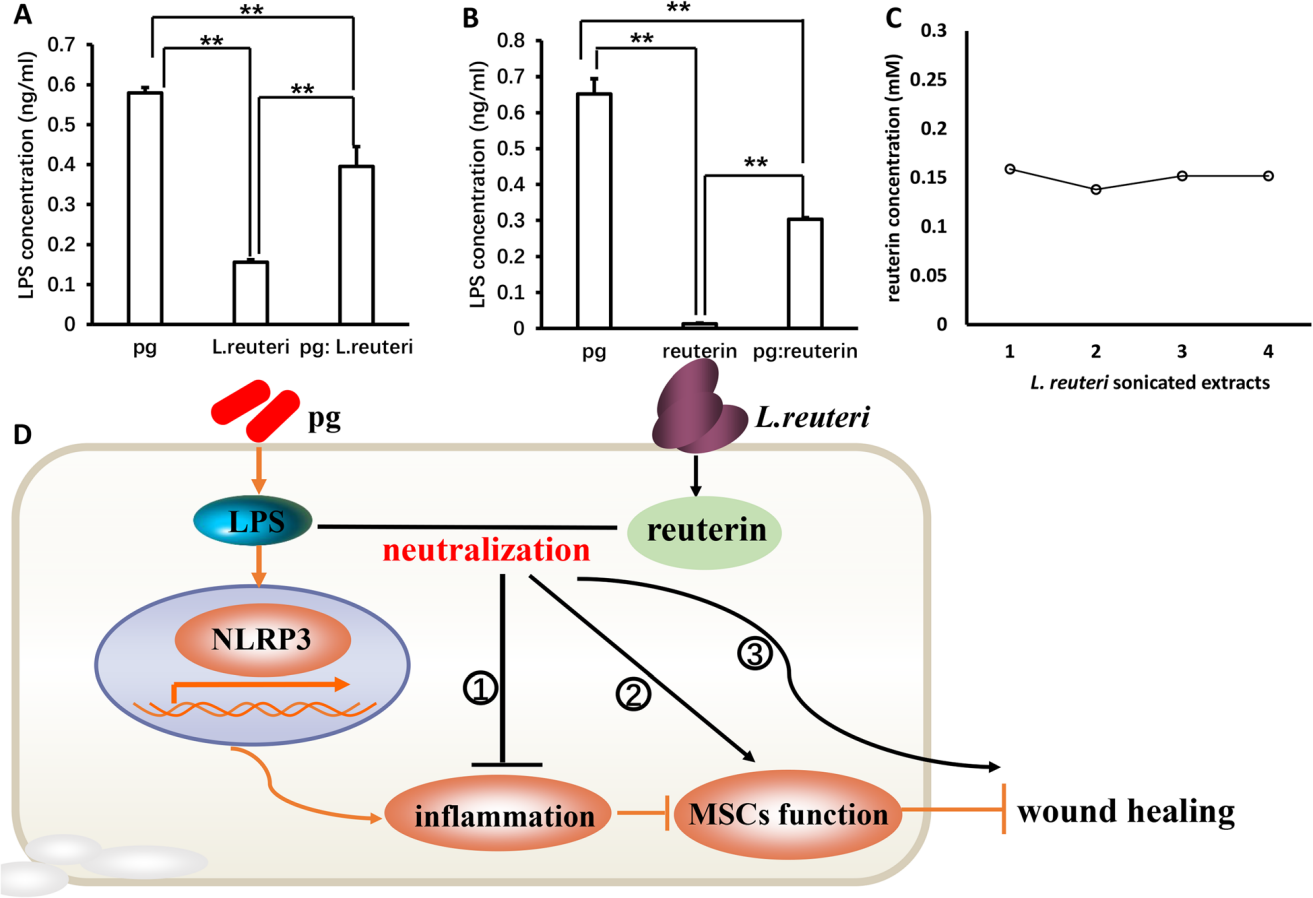
Reuterin maintained the balance of oral pathogenic bacteria and probiotics by neutralizing LPS. **a** ELISA assay results demonstrated that a large amount of LPS existed in *P. gingivalis* sonicated extracts, and the content of LPS was significantly decreased in the mixture of *P. gingivalis* bacteria extract and *L. reuteri* sonicated extract group. **b** ELISA assay results showed that reuterin could neutralize LPS. **c** Chromatography assay results further confirmed that *L. reuteri* sonicated extracts contained reuterin, and reuterin maintained the balance of pathogenic bacteria and probiotics by neutralizing LPS in *P. gingivalis*, thus promoting wound healing (**d**). One-way ANOVA and Student’s *t* test were used to calculate statistical significance. Error bars represent SD (*n* = 3). *P ≤ 0.05; **P ≤ 0.01

It is well known that LPS is the main pathogenic component of gram-negative bacteria, which caused a series of oral diseases such as periodontitis, gingivitis, and inflammatory disease and impaired the functions of MSCs [29–32]. Next, to detect whether bacterial sonicated extracts existed LPS and the interaction between pathogenic bacteria and probiotics, ELISA assay was used to measure the content of LPS; our results demonstrated that a large amount of LPS existed in *P. gingivalis* sonicated extracts; however, the content of LPS was significantly decreased in the mixture of *P. gingivalis* bacteria extract and *L. reuteri* sonicated extract group (Fig. 7a). This suggested that some ingredients could neutralize LPS in *L. reuteri* sonicated extracts. Next, we found that LPS was obviously reduced after *P. gingivalis* bacteria extracts interacted with reuterin (Fig. 7b). Chromatography assay results further confirmed that *L. reuteri* sonicated extracts contained reuterin (Fig. 7c); thus, LPS in *P. gingivalis* could activate *NLRP3* inflammasome resulting in inflammation and inhibit function of MSCs, thereby accelerating MSC dysfunction and delaying wound healing. When pathogenic *P. gingivalis* with *L.reutri* were mixed, reuterin was the effective ingredient in *L. reuteri* which maintained the balance of pathogenic bacteria and probiotics by neutralizing LPS in *P. gingivalis*, thus promoting wound healing (Fig. 7d).

## Discussion

To date, an increasing number of studies have suggested that the balance of oral microbial ecosystem plays an indispensable role in maintaining human health. A shift of oral microbiota may cause both oral and systemic diseases [33, 34]. Our previous study revealed that the imbalance of oral microbiome impaired GMSC proliferation and palatal wound healing. However, the impact of balance of oral microbial ecosystem on MSC function and wound healing, and the mechanisms of interactions between oral bacterial communities remain elusive. In oral cavity, some specific bacteria, such as *P. gingivalis*, can change the proportion of other microorganisms in the ecological niche by changing the environment and are defined as key pathogen [35, 36]. Thus, in this study, we used *P. gingivalis* as the pathogenic state, and the ratio of *P. gingivalis* to *L. reuteri* sonicated extracts was 1 to 0.5 which was used to mimic the balance of oral microbiota; we discovered that 50 μg/ml *P. gingivalis* sonicated extracts significantly inhibited GMSC migration, osteogenic differentiation, and proliferation capacities, and promoted adipogenic differentiation in vitro. However, when the mixture of *L. reuteri* sonicated extracts and *P. gingivalis* bacterial extracts was used to treat GMSCs, we found that the migration, osteogenic and adipogenic differentiation, and cell proliferation potentials of GMSCs could be restored in oral microecological balance. LPS, a specific component of the cell wall of gram-negative bacteria, can induce cascade reaction of immune stimulation and toxic pathophysiological activities in the body [37–39]. Our previous study confirmed that LPS increased in mice with antibiotic treatment, which resulted in delayed wound healing and inhibited the proliferation capacity of GMSCs. In the present study, we found that *P. gingivalis* sonicated extracts contained large amounts of LPS, suggesting that LPS was a key pathogenic factor which could impair MSC function and wound healing. Moreover, we discovered that the content of LPS was significantly reduced after mixing *P. gingivalis* and *L. reuteri* sonicated extracts. *L. reuteri* not only has the main beneficial effect of *Lactobacillus*, it also has the ability to produce a broad spectrum of antimicrobial agent [40–42]. This substance, termed reuterin, has been reported to effectively inhibit the growth of harmful bacteria. Here, high levels of reuterin were tested in *L. reuteri* sonicated extracts, which could neutralize LPS and restore the balance between pathogenic bacteria and probiotics, and reuterin could promote the migration of GMSCs. These findings collectively suggest that reuterin maintained the balance of oral microecology by neutralizing LPS, thus restoring the function of GMSCs.

To further test the impact of the balance of oral microbiota on wound healing, we created the wound healing model in mice after antibiotic treatment. According to macroscopic observation and histopathological photomicrographs, we found that local inoculation of *P. gingivalis* significantly inhibited wound healing; however, inoculation of the mixture of *P. gingivalis* and *L. reuteri* markedly promoted the wound healing process. In addition, histopathological photomicrographs of the wound healing model further indicated that local inoculation of the mixture of *P. gingivalis* and *L. reuteri* accelerated the wound healing in comparison with the *P. gingivalis*-inoculated and untreated groups. These data suggested that the balance of oral microbiota played an important role in promoting wound healing.

Inflammasomes are multiprotein complexes that can recognize a variety of pathogenic microorganisms and stress-related endogenous signaling molecules and play a critical role in innate immune system. As the core of inflammatory response, *NLRP3* inflammasome plays a key role in many diseases, including familial periodic inflammation, type 2 diabetes mellitus, Alzheimer’s disease, and atherosclerosis. It has been reported that *P. gingivalis*-LPS could activate *NLRP3* inflammasome in mouse periodontal ligament fibroblasts and cause chronic periodontitis; however, the relevance of *NLRP3* inflammasome activation and MSC function is far from clear [43, 44]. In this study, we provide evidence that LPS in *P. gingivalis* could activate *NLRP3* inflammasome and inhibit the migration, self-renewal capacity, osteogenic differentiation, and cell proliferation of GMSCs, indicating that *NLRP3* inflammasome activation could accelerate MSC dysfunction and that reuterin is critical for MSC function due to its effect on inhibition of *NLRP3* inflammasome activation.

In summary, our data showed that the balance of oral microbiota can activate the functions of MSCs and that reuterin in *L. reuteri* maintained the balance of oral microecology by neutralizing LPS, thus maintaining the potentials of MSCs and promoting wound healing. Our study shows a link between the balance of oral microecology and wound healing, which suggests the importance of oral microbiome balance in sustaining MSC function and oral homeostasis. However, oral microbes are composed of a variety of bacteria, and we only used the mixture of *P. gingivalis* and *L. reuteri* to mimic the balance of oral microecology in this work. Future investigations will need a wider variety of bacteria to elucidate the effect of oral microbiome balance on oral health.

## Conclusion

In a word, our findings revealed that simulating the balance between oral pathogenic bacteria and probiotics with sonicated extracts from *L. reuteri* and *P. gingivalis* could activate the functions of MSCs and that reuterin in *L. reuteri* maintained the balance of oral microecology by neutralizing LPS in *P. gingivalis*, thus maintaining the homeostasis of MSCs and promoting wound healing. Our findings are based on the concept of the balance of oral microecology, which provides promising ideas and methods for the prevention and treatment of oral diseases, and can be used for reference in other systemic diseases caused by dysfunction of microbiota and MSCs.

## Supplementary information

**Additional file 1: Table S1.** Primers sequences used in the real-time RT-PCR

**Additional file 2: Figure S1.** The balance of oral pathogenic bacteria - *P. gingivalis* and probiotics- *L. reuteri* extracts promoted wound healing in mice. (A) Macroscopic observation showed that wound healing model of palate was established in mice after antibiotics treatment. (B) The local injection of P. gingivalis extracts delayed wound healing after 7 days. (C) The local injection of the mixture of *L. reuteri* extracts and P. gingivalis extracts group promoted wound healing compared with untreated group (D). (E) Quantitative analysis of the unhealed area of wound after inoculation for 7 days respectively. Scale bar: 1 mm. Error bars represent SD (*n* = 6). Red dotted line: original the area of wound, Yellow dotted line: the unhealed area of wound. * *P* ≤ 0.05; ** *P* ≤ 0.01.

**Additional file 3: Figure S2.** The expression of surface markers in GMSCs from C57BL/6 mice. (A, B, C) GMSCs were isolated from mice expressed CD146. Scale bar: 50 μm. (D, E) Flow cytometric analysis results (CD146 positive rate: 97.1%). (F, G, H) GMSCs were from C57BL/6 mice expressed CD90. Scale bar: 50 μm. (I, J) Flow cytometric analysis results (CD90 positive rate: 90.7%). (K, L, M) CD45 were not expressed in GMSCs obtained from mice. Scale bar: 50 μm. (N, O) Flow cytometric analysis results (positive rate: 1.69%). (P, Q, R) GMSCs were from C57BL/6 mice expressed CD44. Scale bar: 50 μm. (S, T) Flow cytometric analysis results (CD44 positive rate: 87.8%). Student’s t-test was utilized for analysis in E, J, O, T. Error bars represent SD (*n* = 3). *P ≤ 0.05; **P ≤ 0.01.

## Data Availability

All data can be attained from corresponding authors.

## Abbreviations

ALP: Alkaline phosphatase
ARS: Alizarin red staining
GAPDH: Glyceraldehyde-3-phosphate dehydrogenase
GMSCs: Gingival mesenchymal stem cells
LPL: Lipoprotein lipase
NANOG: Nanog homebox
OCN: Osteocalcin
OCT4: Octamer-binding transcription factor 4
OPN: Osteopontin
OSX: Osterix
PPAR-γ: Peroxisome proliferater-activated receptor
SOX2: Sex-determining region Y-box 2
