# Supplementary material for: Balanced oral pathogenic bacteria and probiotics promoted wound healing via maintaining mesenchymal stem cell homeostasis
Source: Stem Cell Res Ther. 2020 Feb 14;11:61. doi: 10.1186/s13287-020-1569-2 (PMC7023757; doi:10.1186/s13287-020-1569-2)
Supplement: Supplementary file 1 — Additional file 1: Table S1. Primers sequences used in the real-time RT-PCR [file 13287_2020_1569_MOESM1_ESM.doc]

**Supplementary file: Table 1. Primers sequences used in the real-time RT-PCR**

**Gene Symbol Primer Sequences (5’-3’)**

*GAPDH*-F GAAGATATGGGCACAGGGGA

*GAPDH*-R CAAGAAGATGCGGCTGTCTC

*SOX2*-F CGGCACAGATGCAACCGAT

*SOX2*-R CCGTTCATGTAGGTCTGCG

*OCT4*-F AGAGGATCACCTTGGGGTACA

*OCT4*-R CGAAGCGACAGATGGTGGTC

*NANOG*-F TCTTCCTGGTCCCCACAGTTT

*NANOG*-R GCAAGAATAGTTCTCGGGATGAA

*OCN*-F CAGACAAGTCCCACACAGCA

*OCN*-R CTTGGCATCTGTGAGGTCAG

*OSX*-F TCCCTGGATATGACTCATCCCT

*OSX*-R CCAAGGAGTAGGTGTGTTGCC

*RUNX2*-F GACTGTGGTTACCGTCATGGC

*RUNX2*-R ACTTGGTTTTTCATAACAGCGGA

*OPN*-F AGAGCGGTGAGTCTAAGGAGT

*OPN*-R TGCCCTTTCCGTTGTTGTCC

*NLRP3*-F ATCAACAGGCGAGACCTCTG

*NLRP3*-R GTCCTCCTGGCATACCATAGA
