# Supplementary figures and images for: Balanced oral pathogenic bacteria and probiotics promoted wound healing via maintaining mesenchymal stem cell homeostasis
Source: Stem Cell Res Ther. 2020 Feb 14;11:61. doi: 10.1186/s13287-020-1569-2 (PMC7023757; doi:10.1186/s13287-020-1569-2)

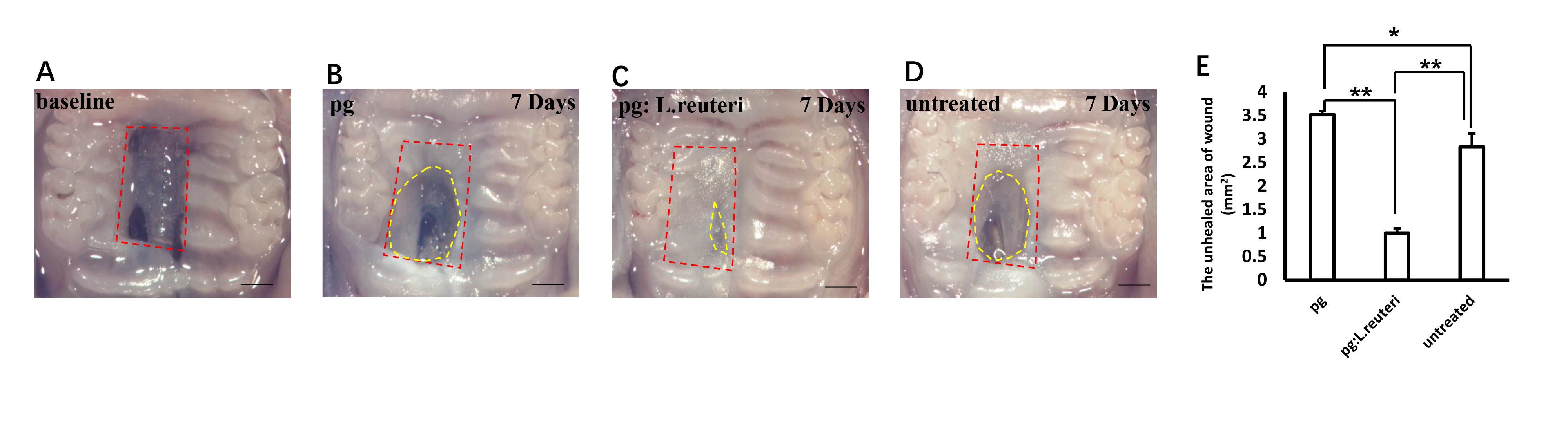

Supplement: Supplementary file 2 — Additional file 2: Figure S1. The balance of oral pathogenic bacteria - P. gingivalis and probiotics- L. reuteri extracts promoted wound healing in mice. (A) Macroscopic observation showed that wound healing model of palate was established in mice after antibiotics treatment. (B) The local injection of P. gingivalis extracts delayed wound healing after 7 days. (C) The local injection of the mixture of L. reuteri extracts and P. gingivalis extracts group promoted wound healing compared with untreated group (D). (E) Quantitative analysis of the unhealed area of wound after inoculation for 7 days respectively. Scale bar: 1 mm. Error bars represent SD (n = 6). Red dotted line: original the area of wound, Yellow dotted line: the unhealed area of wound. * P ≤ 0.05; ** P ≤ 0.01. [file 13287_2020_1569_MOESM2_ESM.tif]

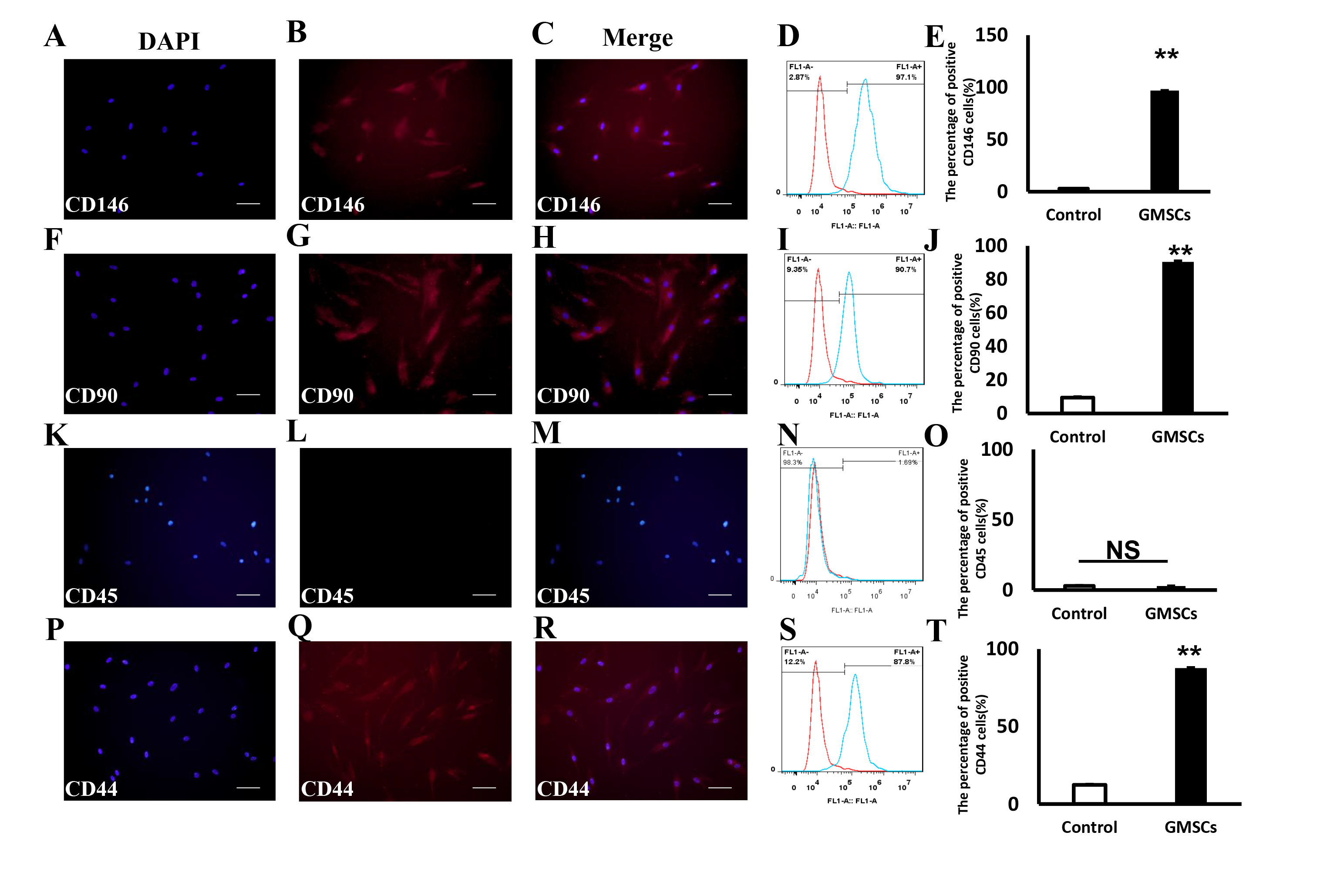

Supplement: Supplementary file 3 — Additional file 3: Figure S2. The expression of surface markers in GMSCs from C57BL/6 mice. (A, B, C) GMSCs were isolated from mice expressed CD146. Scale bar: 50 μm. (D, E) Flow cytometric analysis results (CD146 positive rate: 97.1%). (F, G, H) GMSCs were from C57BL/6 mice expressed CD90. Scale bar: 50 μm. (I, J) Flow cytometric analysis results (CD90 positive rate: 90.7%). (K, L, M) CD45 were not expressed in GMSCs obtained from mice. Scale bar: 50 μm. (N, O) Flow cytometric analysis results (positive rate: 1.69%). (P, Q, R) GMSCs were from C57BL/6 mice expressed CD44. Scale bar: 50 μm. (S, T) Flow cytometric analysis results (CD44 positive rate: 87.8%). Student’s t-test was utilized for analysis in E, J, O, T. Error bars represent SD (n = 3). *P ≤ 0.05; **P ≤ 0.01. [file 13287_2020_1569_MOESM3_ESM.tif]
